# Diagnosis and Management of Adult Tuberculosis Patients Admitted to a Rural Hospital in Ethiopia

**DOI:** 10.7759/cureus.35519

**Published:** 2023-02-27

**Authors:** Belén Comeche, Mario Pérez-Butragueño, Miguel Górgolas, José-Manuel Ramos-Rincón

**Affiliations:** 1 Department of Medicine and Pediatrics, Gambo General Rural Hospital, Gambo, ETH; 2 Department of Internal Medicine, Hospital Universitario Infanta Leonor, Madrid, ESP; 3 Department of Pediatrics, Hospital Universitario Infanta Leonor, Madrid, ESP; 4 Division of Infectious Diseases, Hospital Universitario Fundación Jimenez Díaz, Autonomous University Madrid, Madrid, ESP; 5 Department of Internal Medicine, Hospital Universitario General de Alicante-Instituto de Investigación Sanitaria y Biomédica de Alicante (ISABIAL) and Miguel Hernández University of Elche, Alicante, ESP

**Keywords:** adult, rural hospital, management, diagnosis, ethiopia, tuberculosis

## Abstract

Background

Ethiopia is one of the countries in the world with the highest rate of tuberculosis (TB). The aim of this study is to describe the characteristics of the patients with TB admitted to a rural hospital in Ethiopia in terms of both diagnosis and clinical management.

Methods

A retrospective descriptive observational study was conducted. Data were collected from patients older than 13 years who were admitted to the Gambo General Hospital for TB between May 2016 and September 2017. The variables studied were age, sex, symptoms, human immunodeficiency virus (HIV) serology, nutritional status, presence of anemia, chest x-ray or other complementary tests, type of diagnosis (smear microscopy, Xpert MTB-RIF (Cepheid, Sunnyvale, California, USA), or clinical diagnosis), treatment received, outcome, and days of admission.

Results

One hundred eighty-six patients, aged 13 years and older, were admitted to the TB unit. About 51.6% were female, and the median age was 35 years (interquartile range (IQR) 25-50). Cough was the most frequent symptom on admission (88.7%), and contact with a TB patient was only recognized by 22 patients (11.8%). HIV serology was performed in 148 patients (79.6%); seven were positive (4.7%). About 69.3% met the criteria for malnutrition (body mass index (BMI) <18.5). Most patients, 173 (93%), presented with pulmonary TB and were new cases (94.1%). Patients were diagnosed by clinical parameters in 75% of cases. Smear microscopy was performed in 148 patients, of which 46 (31.1%) were positive, and Xpert MTB-RIF results were only obtained in 16 patients, of which 6 (37.5%) were positive. Chest x-rays were performed in most patients (71%) and were suggestive of TB in 111 (84.1%). The average length of hospital stay was 32 days (confidence interval (CI) 13-50.5). Women tend to be younger than men, have more extrapulmonary TB, and were admitted longer. Nineteen patients died during admission (10.2%). Patients who die were more frequently malnourished (92.9% of those who die were malnourished compared to 67.1% of those who did not die, p = 0.036), tend to be admitted for a shorter time than the survivors and receive more concomitant antibiotic treatment.

Conclusions

In this rural Ethiopian setting, patients admitted to the hospital for TB are often malnourished (67.1%), the main presentation is pulmonary, mortality is one in 10 admissions and very often receive antibiotics in association with TB treatment (40%).

## Introduction

Ethiopia is a country with a significant burden of poverty, and its economy is still based primarily on agriculture and livestock, with more than 70% of the population living in rural locations [1]. It is one of the countries in the world with the highest rate of tuberculosis (TB) and human immunodeficiency virus (HIV)-TB co-infection [2]. For these reasons, TB is a main concern for the government, which has established protocols for TB management, including diagnostic and therapeutic processes [3].

Gambo General Hospital (GGH) is a rural (tertiary) facility located about 250 km south of the capital that has a unit dedicated to in-patients with TB. It also has a laboratory for the microbiological diagnosis of TB by fluorescence microscopy as well as a basic radiology unit. Since 2015, it is possible to send samples﻿ to the reference laboratory for the Xpert MTB/RIF (Cepheid, Sunnyvale, California, USA) assay following national protocols. The aim of this study was to describe the characteristics of the patients with TB admitted to this hospital in terms of both diagnosis and clinical management.

## Materials and methods

A retrospective descriptive observational study was conducted. Data were collected from patients older than 13 years who were admitted to the GGH for TB between May 2016 and September 2017. The inclusion criteria were patients older than 13 years with a diagnosis of TB who were admitted to the GGH during the study period. The rest of the admitted patients were excluded.

The variables studied were age, sex, symptoms, HIV serology, nutritional status, presence of anemia, chest x-ray or other complementary tests, type of diagnosis (smear microscopy, Xpert MTB-RIF, or clinical diagnosis), treatment received, outcome, and days of admission.

All data collected were double-checked for completeness. They were entered into a database (Microsoft Excel version 16.70, Redmond, Washington, USA) and exported to the statistical program SPSS (IBM SPSS version 24, Armonk, New York, USA) for analysis. Continuous variables were expressed as the median and interquartile range (IQR), and qualitative variables as value and percentage. The Chi-square test and Mann-Whitney U test were used to compare data where appropriate, interpreting results using odds ratios (OR) and 95% confidence intervals (95% CI). A bivariate analysis was performed to identify variables associated with the outcome of death and with TB by gender, according to the chronological and anatomical classification of TB patients.

The Research Committee and Ethics Review Board of the Gambo General Rural Hospital, Ethiopia (Code GGRH-241-18) and the Research Ethics Committee of the Fundación Hospital Universitario Jiménez Díaz, Spain (Code EO187-18_FJD) approved the study. Due to its retrospective nature, the need for informed consent from patients, parents, or legal guardians was waived.

## Results

Clinical characteristics

Between May 2016 and September 2017, 186 patients aged 13 years and older were admitted to the TB unit of GGH. Of these, 90 were male (48.4%) and 96 were female (51.6%), with a median age of 35 years (IQR 25-50). Cough was the most frequent symptom on admission (88.7%), and contact with a TB patient was only recognized by 22 patients (11.8%). Human immunodeficiency virus (HIV) serology was performed in 148 patients (79.6%); seven were positive (4.7%) (Table 1).

**Table 1:** Clinical characteristics and diagnostic process (N = 186). n/N: number of cases/total of cases; %: percentage; IQR: interquartile range; TB: tuberculosis; HIV: human immunodeficiency virus; BMI: body mass index; MUAC: mid-upper arm circumference.

Variables	
Age, median (IQR)	35 (25-50)
Female, ﻿n (%)	96 (51.6)
Symptoms	
Cough, ﻿n (%)	165 (88.7)
Fever, ﻿n (%)	33 (17.7)
Adenopathies, ﻿n (%)	10 (5.4)
Contact TB, ﻿n (%)	22 (11.8)
HIV test, n (%)	148 (79.6)
Positive, ﻿n/N (%)	7/148 (4.7)
Negative, ﻿n/N (%)	141/148 (95.2)
Nutritional status	
Malnutrition by BMI, ﻿n/N (%)	115/166 (69.3)
Malnutrition by MUAC, ﻿n/N (%)	100/120 (83.3)
Type of TB	
Pulmonary TB, ﻿n (%)	173 (93.0)
Extrapulmonary TB, ﻿n (%)	13 (7.0)
Case type	
New case, ﻿n (%)	175 (94.1)
Pre-treated, ﻿n (%)	11 (5.9)
Sputum smear microscopy, n (%)	148 (79.5)
Positive, ﻿n/N (%)	46/148 (31.1)
Negative, ﻿n/N (%)	102/148 (68.9)
Xpert MTB-RIF, n (%)	16 (8.6)
Positive, ﻿n/N (%)	6/16 (37.5)
Negative, ﻿n/N (%)	10/16 (62.5)
Chest x-ray, ﻿n (%)	132 (71.0)
Suggestive of TB, ﻿n/N (%)	128/132 (97.0)
Not suggestive of TB, ﻿n/N (%)	4/132 (3.0)

Nutritional status was assessed by body mass index (BMI) in 166 patients, of whom 115 (69.3%) met the criteria for malnutrition (BMI<18.5). Likewise, mid-upper arm circumference (MUAC) was also used in 120 patients, where 100 (83.3%) met malnutrition criteria (<22 cm for women and <23 cm for men).

Regarding the location of TB and following the 2012 and 2017 national protocols, most patients, 173 (93%), presented with pulmonary tuberculosis (PTB) (any patient with pulmonary infiltrates). Most of the patients were new cases, 175 (94.1%), compared to only 11 patients (5.9%) classified as pre-treated (according to national guidelines that consider pre-treated those patients who have received previous treatment for at least one month).

Diagnostic process

Patients were diagnosed by clinical parameters (compatible clinical history or chest x-ray) in 75% (139/186) of cases and 25% (47/186) by smear or Xpert. Smear microscopy was performed in 148 patients, of which 46 (31.1%) were positive, and Xpert MTB-RIF results were only obtained in 16 patients, of which 6 (37.5%) were positive (Table 1).

Chest x-rays were performed in most patients (71%), and they were suggestive of TB in 111 (84.1%), with patterns such as uni- or bilateral infiltrates, miliary pattern, and cavities. In 22 (16.6%) patients, no compatible patterns were found (normal, cardiomegaly, or pneumothorax).

Outcomes

The average length of hospital stay was 32 days (confidence interval 95% (95%CI) 13-50.5). One hundred and fifty patients (80.6%) were discharged for further follow-up at their health posts; 19 patients died during admission (10.2%); 10 cases (5.4%) were transferred to another hospital for several reasons; and seven patients (3.8%) requested voluntary discharge or disappeared from the hospital without prior notice (Table 2). There were no patients readmitted.

**Table 2:** Outcomes and treatment. CI: confidence interval, H: isoniazid, R: rifampicin, Z: pyrazinamide, E: ethambutol, NSAIDs: non-steroidal anti-inflammatory drugs, HIV: human immunodeficiency virus.

Variables	
Days of admission, mean (CI)	32 (13-50.5)
Outcomes	
Hospital discharge, n (%)	150 (80.6)
Exitus, n (%)	19 (10.2)
Transferred to other hospital, n (%)	10 (5.4)
Voluntary discharge/hospital leakage, n (%)	7 (3.8)
Tuberculostatic treatment	
HRZE, n (%)	174 (93.5)
HR, n (%)	11 (5.9)
None, n (%)	1 (0.5)
Antibiotics, n (%)	81 (43.5)
One antibiotic, n (%)	57 (30.1)
Two, n (%)	20 (10.8)
Three, n (%)	3 (1.6)
Four, n (%)	1 (0.5)
Other antimicrobials	
Fluconazole, n (%)	2 (1.1)
Anthelmintics, n (%)	6 (3.2)
HIV antiviral treatment, n (%)	7 (4.7)
Other drugs	
Vitamin complexes, n (%)	43 (23.1)
Corticosteroids, n (%)	7 (3.8)
NSAIDs, n (%)	44 (23.7)
Diuretics, n (%)	18 (9.7)

Treatment

Most are treated with four TB drugs (174 patients, 93.5%) as admission coincides with the intensive treatment phase; there were 11 patients who received two drugs because they were in the second phase of treatment (5.9%), and only one did not receive any drugs at all. Other concomitantly administered drugs were antibiotics in 81 patients (43.5%), the most frequent being ceftriaxone in 72 patients (38.7%), vitamin complexes in 43 patients (23.1%), corticosteroids in seven patients (3.8%), non-steroidal anti-inflammatory drugs (NSAIDs) in 44 patients (23.7%), diuretics in 18 patients (9.7%), and transfusions in three patients (1.6%).

Analysis by gender and outcome

Women have a greater tendency to present extrapulmonary TB (EPTB) (nine women vs. four men, p = 0.188) and especially lymphadenopathies (eight women vs. two men, p = 0.075). They tend to be younger than men (mean age: men 40.9 years, women 35.1 years, p = 0.011). A significant difference was also observed in the duration of admission, with women staying longer (36.3 days in women vs. 27.8 days in men, p = 0.009) (Table 3).

**Table 3:** Hospitalization data by gender and outcome. n: number of cases; %: percentage; TB: tuberculosis; PTB: pulmonary; TB, EPTB: extrapulmonary TB; H: isoniazid, R: rifampicin, Z: pyrazinamide, E: ethambutol; MUAC: mid-upper arm circumference; BMI: body mass index.

	Men, n = 90 (48.4%)	Women, n = 96 (51.6%)	p-value	Exitus, n = 19 (10.2%)	Alive, n = 167 (89.8%)	p-value
Clinical and demographic variables						
Age, mean	40.9	35.1	0.011	43.7	37.3	0.163
Cough, n (%)	83 (92.2)	82 (85.4)	-	16 (84.2)	149 (89.2)	0.365
Fever, n (%)	18 (20.0)	15 (15.6)	-	5 (26.3)	28 (16.8)	0.229
Adenopathies, n (%)	2 (2.5)	8 (8.9)	0.075	0	10 (6,6)	0.314
Nutritional status (malnutrition)						
MUAC, n (%)	55 (61.1)	60 (62.5)	-	8 (88.9)	92 (82.9)	0.538
BMI, n (%)	45 (50.0)	55 (57.3)	-	13 (92.9)	102 (67.1)	0.036
TB location						
PTB, n (%)	86 (95.6)	87 (90.6)	0.188	19 (100)	154 (92.2)	0.207
EPTB, n (%)	4 (4.4)	9 (9.4)	-	2 (10.5)	9 (5.4)	-
Type of TB						
New, n (%)	83 (92.2)	92 (95.8)	-	17 (89.5)	158 (94.6)	0.312
Pre-treated, n (%)	7 (7.8)	4 (4.2)	-	2 (10.5)	9 (5.4)	-
Outcome and treatment						
Days admission, mean	27.8	36.3	0.009	12.3	34.5	<0.0001
Discharged, n (%)	67 (74.4)	83 (86.5)	-			
Exitus, n (%)	12 (13.3)	7 (7.3)	0.132			
Transfer, n (%)	7 (7.8)	3 (3.1)	-			
Voluntary discharge, n (%)	4 (4.4)	3 (3.1)	-			
Tuberculostatic treatment						
HRZE, n (%)	85 (94.5)	89 (92.7)	-	18 (94,7)	156 (93,4)	0.824
HR, n (%)	4 (4.4)	7 (7.3)	-	0	11 (6,6)	-
No treatment, n (%)	1 (1.1)	0	-	1 (5.3)	0	-
Antibiotics during admission						
No, n (%)	50 (55.6)	55 (57.3)	-	5 (26.3)	100 (59.9)	0.0052
Yes, n (%)	40 (44.4)	41 (42.7)	-	14 (73.7)	67 (40.1)	-
Other treatments: diuretics, NSAIDs, corticosteroids, and vitamin supplements						
No, n (%)	52 (57.8)	56 (58.3)	-	11 (57.9)	97 (58.1)	0,987
Yes, n (%)	38 (42.2)	40 (41.7)	-	8 (42.1)	70 (41.9)	-

Patients who die are more frequently malnourished, according to BMI criteria (92.9% of those who die are malnourished compared to 67.1% of those who do not die, p = 0.036). They also tend to be admitted for a shorter time than the survivors (mean 12 days compared to 34 days for those who do not die, p<0.0001) and receive more concomitant antibiotic treatment (73.7% vs. 40.1%, p = 0.005).

According to the temporal classification of patients (Table 4), pre-treated patients are older (48 years vs. 37.3 years, p = 0.075), although without statistical significance. Pre-treated patients are more likely to undergo Xpert (36.4% vs. 10.3%, p = 0.009) as well as more likely to have a positive Xpert result (18.2% vs. 2.3%, p = 0.004). In addition, these patients have a longer stay in the hospital (52.1 days vs. 31 days, p = 0.005) and receive more antibiotics than new patients (81.8% vs. 41.1%, p = 0.008).

**Table 4:** Comparison between new and pre-treated patients. n: number of cases; %: percentage; HIV: human immunodeficiency virus.

	New n = 175 (94.1%)	Pre-treated n = 11 (5.9%)	p-value
Age, mean (z score)	37.3 (15.3)	48 (19.5)	0.075
HIV			
Positive, n (%)	7 (4%)	0	0.5
Negative, n (%)	135 (77.1)	6 (54.5)	0.09
Unknown, n (%)	33 (18.9)	5 (45.5)	0.034
Xpert result			
Not done, n (%)	157 (89.7)	7 (63.6)	0.009
Done, n (%)	18 (10.3)	4 (36.4)	-
Negative, n (%)	9 (5.1)	1 (9.1)	0.573
Positive, n (%)	4 (2.3)	2 (18.2)	0.004
Lost, n (%)	5 (2.9)	1 (9.1)	0.256
Length of stay, mean (z score)	31 (21.3)	52.1 (24.2)	0.005
Exitus	19	0	0.249
Antibiotics			
Yes, n (%)	72 (41.1)	9 (81.8)	0.008
No, n (%)	103 (58.9)	2 (18.2)	-

## Discussion

There are few published data on TB patients admitted to hospitals in Ethiopia. Most of the publications are on patients co-infected with HIV or with multidrug-resistant TB (MDR-TB). There is also scientific literature on patients in directly observed treatment (DOT) programs in Ethiopia [4,5]. Our population has a similar sex and age distribution to that observed in other studies (50% male-to-female ratio, 28 to 37 years on average) [5-7].

Our HIV-positive rate is 4.7%, which is lower than in other series in Oromia (where about 30% of TB patients are HIV positive) and in other areas of Ethiopia, with rates around 19-26%. In addition, in our study, the HIV test was not performed in 20.4% of the patients, while in other studies it was not performed in only 4% [8-10]. Perhaps the difference with these studies with higher HIV rates, is that our population is fundamentally rural, compared to those studies with a high percentage of the urban population.

Regarding TB location, most of the patients in our study were classified as PTB (93%), compared to 7% of EPTB patients. These rates are much higher than those published in other series, both in general hospitals, referral hospitals, and in patients in the DOTs program (14-57%) [6,9,11,12]. Ethiopia is known to be a country with a high rate of EPTB; however, the exact risk factors associated with this are not known or the results are inconclusive [13]. Patients with EPTB in our series are younger than those presented by Berg et al. [13] and tend to be females; we have not found any difference in HIV serostatus, although EPTB has been described as a risk factor for HIV [14].

The percentage of pre-treated patients in our series is low compared to other studies in referral hospitals (5.9% vs. 12.1%) [15], but similar to the rates in general hospitals [16]. This is probably influenced by the fact that GGH is not a referral center in the area, and, therefore, some of the pre-treated patients are referred from the health posts to the referral hospital in the area and not to GGH. In our series, new patients are younger, have shorter hospital stays and receive fewer antibiotics than the pre-treated ones. This may be because patients who have been treated before (according to the definition in the guidelines, those who have received at least one month of anti-tuberculosis treatment) come to the hospital in worse conditions than those who are diagnosed for the first time, although we have not found any difference in the rate of exitus.

We obtained a mortality rate of 10.2%, but in different studies [17], it varies greatly [18]. There is no significant difference in terms of mortality between those who receive the diagnosis during admission or before admission. The reasons for hospitalization of the latter are mainly clinical worsening, intolerance to drugs or poor compliance to treatment. Patients who die spend fewer days in hospitals than those who survive. The reason for this could be that they are admitted in a worse clinical situation, and their evolution is also worse. In addition, patients who die receive more antibiotics than those who survive, probably because of suspected superinfections, which could be the cause of death.

We have found a high rate of malnutrition (61% of patients). Poor nutritional status is known to be closely related to TB. Some Ethiopian series describe rates of around 28-40% [19,20], while others, such as Hussien et al. and Ephrem et al., show results similar to ours [21,22]. The differences may lie in the type of patient studied, as the series in which patients are newly diagnosed show higher rates of malnutrition compared to those in which patients are included at various stages of follow-up. In our study, patients were recently diagnosed and therefore at the beginning of therapy, so it seems reasonable to think that malnutrition could be one of the major determinants for the patient being more symptomatic and requiring healthcare.

Also relevant is the number of antibiotics used concomitantly. About 43% of patients received antibiotics along with anti-tuberculosis treatment, and two different antibiotics were used in 10% of them. It is difficult to compare these data with other series, as there are no similar data published. The WHO has published guidelines for the appropriate use of antibiotics, as it has been established that 30% of the antibiotics administered could be used inappropriately [23]. In Ethiopia, there is considerable published data on the use of antibiotics in primary care, with figures higher than recommended in the WHO guidelines [23-25]. There are no specific studies on antibiotic prescriptions for inpatients admitted with TB; however, there are studies that found antibiotic use in 62-74% of the patients admitted for any reason [26-28], and specifically, with ceftriaxone in almost 30% of all [29]. Our data, although lower than those, reflect a widespread use of antibiotics, probably unnecessary in some cases due to the diagnosis of TB.

Our study has limitations related to the difficulty of obtaining data from non-digitized medical records in low-income countries. However, this study is unique because it describes the population admitted to a TB unit in a rural hospital and its clinical characteristics, as well as the diagnostic process, treatment provided, and outcome. Knowing these peculiarities can help to implement specific programs for these hospitalized patients, reinforcing nutritional treatment and adjusting and improving the use of antibiotics.

## Conclusions

In this rural Ethiopian setting, patients admitted to the hospital for TB are often malnourished (67.1%), the main presentation is pulmonary, mortality is one in 10 admissions, and they very often receive antibiotics in association with TB treatment (40%). Knowing these peculiarities can help to implement specific programs for these hospitalized patients, reinforcing nutritional treatment and adjusting and improving the use of antibiotics (antibiotic stewardship).
